# Ultrasound- and fluoroscopy-guided hip aspiration before revision total hip arthroplasty: procedural outcomes and diagnostic accuracy in sequential cohorts

**DOI:** 10.2340/17453674.2026.46931

**Published:** 2026-10-08

**Authors:** Thomas J A VAN SCHAIK, Kevin MAY, Gerjon HANNINK, Job L C VAN SUSANTE, Jon H M GOOSEN, H W Bart SCHREUDER, Wim H C RIJNEN

**Affiliations:** 1 Department of Orthopaedic Surgery, Radboudumc, Nijmegen; 2 Department of Orthopaedic Surgery, Rijnstate Hospital, Arnhem; 3 Department of Orthopaedic Surgery, Sint Maartenskliniek, Nijmegen; 4 Department of Medical Imaging, Radboudumc, Nijmegen, The Netherlands

## Abstract

**Background and purpose:**

Hip aspiration remains essential in the diagnostic work-up of a painful total hip arthroplasty (THA) to assess for periprosthetic joint infection (PJI). In 2020, our institution transitioned from fluoroscopy-guided to ultrasound-guided aspiration. Our study aimed to describe dry-tap proportions, aspirated volumes, and complications, and explore diagnostic accuracy in sequential cohorts.

**Methods:**

This retrospective single-center study included 117 fluoroscopy-guided aspirations (December 2013–June 2020) and 90 ultrasound-guided aspirations (April 2020–March 2024) before revision THA. Procedural outcomes defined as dry taps, aspirated volumes, and complications within 14 days were assessed in the full cohorts, and diagnostic accuracy in procedures with a successful aspiration. Ultrasound-guided biopsy was performed in 39 patients.

**Results:**

The prevalence of PJI was 54/117 (46%) in the fluoroscopy cohort and 25/90 (28%) in the ultrasound cohort (difference −18 percentage points, 95% confidence interval [CI] −31 to −5). Dry taps occurred in 38/117 (32%) and 31/90 (34%) procedures, corresponding to an absolute difference of 2 percentage points (CI –11 to 16). Median aspirated volumes were 5 mL in the fluoroscopy cohort and 4 mL in the ultrasound cohort. No complications were recorded in the fluoroscopy cohort; 2 occurred in the ultrasound cohort, both among patients who underwent biopsy. Sensitivity was 16/34 (47%, CI 30–65) for fluoroscopy and 8/13 (62%, CI 32–86) for ultrasound; specificity was 41/44 (93%, CI 81–99) and 37/40 (93%, CI 80–98).

**Conclusion:**

Dry-tap proportions were similar in these sequential cohorts. 2 complications were recorded, both in patients who underwent biopsy. Specificity was high, whereas sensitivity could be assessed only in patients with a successful aspiration and available culture results, resulting in less precise estimates. The findings do not establish equivalence or superiority.

Diagnosing periprosthetic joint infection (PJI) remains a challenge for orthopedic surgeons. The diagnosis is based on criteria that include clinical features, laboratory tests of peripheral blood and synovial fluid, histological evaluation of periprosthetic tissue, and intraoperative findings [1,2]. In addition, sonication fluid cultures have been proposed as a complementary diagnostic method to intraoperative tissue culture sampling for the diagnosis of PJI [3]. Aspiration of synovial fluid remains a recommended diagnostic modality in the preoperative work-up of a painful total hip arthroplasty (THA) to evaluate for PJI [4].

Fluoroscopy has traditionally been used to perform aspiration of a THA [5]. As practices vary, fluoroscopy may be performed in suites at the radiology department or in the operating room by an orthopedic surgeon. Ultrasound-guided hip aspiration offers several advantages over fluoroscopy-guided methods. It avoids radiation exposure and the need for specialized fluoroscopy suites or operating room capacity, which is often limited and expensive. Real-time ultrasound visualization of the joint facilitates targeted aspiration and may help reduce the number of dry taps [6,7]. Also, this approach allows for the collection of targeted synovial tissue samples during the same session, offering an additional opportunity for diagnostic assessment [8]. Additionally, it may result in greater patient satisfaction due to a less painful procedure [9].

In 2020, our institution changed its standard approach from fluoroscopy-guided to ultrasound-guided hip aspiration. The potential advantages suggested that the transition to ultrasound-guided aspiration might reduce dry taps, although its effect on diagnostic accuracy remained uncertain. The aim of our exploratory study was to describe dry-tap proportions, aspirated synovial fluid volumes, and complications documented within 14 days, and to explore diagnostic accuracy across sequential real-world cohorts undergoing ultrasound-guided or fluoroscopy-guided hip aspiration. As a secondary exploratory objective, we described the diagnostic accuracy after ultrasound-guided biopsy.

## Methods

### Study design and participants

This retrospective single-center cohort study was performed at a university medical center. All patients with a THA who underwent a diagnostic synovial aspiration and subsequently underwent revision THA were eligible for inclusion. In 2020, fluoroscopy-guided hip aspiration was replaced by ultrasound-guided aspiration as the standard diagnostic approach. 2 distinct time periods were analyzed: (i) fluoroscopy-guided aspirations performed between December 2013 and June 2020, and (ii) ultrasound-guided aspirations performed between April 2020 and March 2024. The periods overlapped during the institutional transition from April to June 2020; no patient underwent both techniques. Ultrasound-guided synovial biopsy was performed after a dry tap. In selected cases, it was also performed after a successful aspiration if the orthopedic surgeon considered additional diagnostic information clinically relevant.

The study is reported according to GRRAS guidelines [10].

### Technique of fluoroscopy-guided hip aspiration

All procedures were performed by an orthopedic surgeon in the operating theatre with the patient in supine position. Following sterile skin preparation with chlorhexidine and draping, a 15 cm 18G spinal needle was advanced into the prosthetic joint space under fluoroscopic guidance. Synovial fluid was aspirated, and the total aspirated volume was documented. No saline lavage was performed in case of a dry tap, and the procedure was terminated.

### Technique of ultrasound-guided hip aspiration

All procedures were performed by an experienced musculoskeletal radiology physician assistant with the patient in supine position. Under ultrasound guidance, the anterior hip joint capsule and periprosthetic space were systematically evaluated for synovitis and fluid collections. After sterile skin preparation with chlorhexidine and draping, local anesthesia was administered from the skin to the deep fascia using 1% lidocaine under continuous ultrasound guidance. A 61 mm 16G coaxial support needle was advanced into a stable subfascial position, through which a 20 cm 18G trocar needle was introduced into the prosthetic joint space. Joint aspiration was then performed, and the total aspirated volume was recorded. In the case of a dry tap, aspiration was attempted after repositioning of the needle or flexion of the hip, without the use of saline lavage.

### Technique of ultrasound-guided synovial tissue biopsy

Using the pre-positioned 61 mm 16G coaxial needle, 5 biopsies were obtained with a 16 cm 18G biopsy needle. When safely achievable, a core length of 2.5 cm was targeted, and different accessible joint quadrants were sampled to reduce sampling error due to tissue heterogeneity. Each specimen was transferred to a separate sterile container using a new sterile scalpel blade or needle. Biopsy cultures were considered positive if the same microorganism was isolated from at least 2 of the 5 specimens.

### Specimen handling

Aspirated synovial fluid was divided between a standard 10 mL EDTA tube, with a minimum of 1 mL fluid in the tube, and the remaining fluid retained in a 10 mL Luer-lock syringe with a sterile cap (CombiLock). The EDTA tubes were sent to the clinical laboratory for hemocytometry. The syringe and synovial tissue biopsy containers were sent to the microbiology department for culture.

### Intraoperative procedures and reference standard

All revision procedures were performed according to a standardized protocol. Patients were placed in the lateral decubitus position, after which the surgical site was disinfected with 0.5% chlorhexidine. A posterolateral approach was used in all cases. Six periprosthetic tissue samples were systematically collected for microbiological analysis and immediately sent to the microbiology laboratory. PJI was classified after revision surgery using the European Bone and Joint Infection Society (EBJIS) definition and served as the reference standard [2].

### Data collection and outcomes

Recorded baseline characteristics were age, sex, body mass index (BMI), and reason for revision. Procedural outcomes were dry tap, aspirated volume, ultrasound-guided biopsy, and complications documented within 14 days. For the diagnostic accuracy analysis, aspiration culture was the index test, and postoperative EBJIS classification was the reference standard.

### Statistics

2 independent overlapping cohorts were analyzed: patients undergoing fluoroscopy-guided aspiration (2013–2020) and patients undergoing ultrasound-guided aspiration with selective synovial biopsy (2020–2024).

Baseline characteristics were summarized as medians with interquartile ranges (IQR) for continuous variables and frequencies and percentages for categorical variables. No formal statistical testing or standardized mean differences were calculated.

Procedural outcomes included dry tap rates, aspirated volumes, and complications, summarized descriptively by cohort with exploratory between-cohort differences and 95% confidence intervals (CI). Binomial 95% CIs for proportions were calculated using the Wilson method, and 95% CIs for between-cohort differences in proportions were calculated using the Newcombe–Wilson method. Aspirated synovial fluid volumes were summarized by cohort using medians and IQRs, with between-cohort differences estimated using the Hodges–Lehmann method and corresponding 95% CI.

Diagnostic performance was assessed using 2 x 2 contingency tables with the EBJIS definition of periprosthetic joint infection as the reference standard [2]. Sensitivity and specificity were calculated separately for each cohort. Importantly, diagnostic accuracy analyses were restricted to cases with successful aspiration (i.e., excluding dry taps and laboratory sampling errors), as aspiration-based microbiological results were available only in these patients. All estimates were reported with CIs.

Sensitivity and specificity, as well as between-cohort differences, were reported with CIs using the Wilson and Newcombe–Wilson methods, respectively. No formal hypothesis testing was performed; these comparisons were considered exploratory and were interpreted cautiously given the non-contemporaneous study design and potential differences in disease spectrum between cohorts.

As a sensitivity analysis, diagnostic accuracy was recalculated under 2 scenarios in which dry taps and laboratory sampling errors were classified as (a) test-positive and (b) test-negative, to assess the potential impact of missing microbiology results on diagnostic performance estimates.

As a secondary exploratory analysis, the diagnostic performance of ultrasound-guided synovial tissue biopsy was described using 2 x 2 contingency tables against the predefined reference standard. Sensitivity and specificity were calculated with CIs using the Wilson method. Biopsy findings were summarized overall (irrespective of whether biopsy was performed following a dry tap or after successful aspiration).

Analyses were performed using R (version 4.6.0; R Foundation for Statistical Computing, Vienna, Austria).

### Ethics, registration, data sharing plan, funding, and disclosures

This study was approved by the local institutional review board (approval number 2022-16074). Owing to the retrospective design and use of routinely collected clinical data, the requirement for informed consent was waived. The data supporting the findings is available from the corresponding author upon reasonable request and in accordance with institutional and ethical regulations. This research received no specific grant from any funding agency in the public, commercial, or not-for-profit sectors. Complete disclosure of interest forms according to ICMJE are available on the article page, doi: 10.2340/17453674.2026.46931

## Results

### Study cohorts

The study included 117 fluoroscopy-guided and 90 ultrasound-guided aspirations, all followed by revision THA. Baseline age, sex, BMI, and reason for revision were broadly similar (Table 1). PJI after revision surgery was classified in 54/117 (46%) patients in the fluoroscopy cohort and 25/90 (28%) in the ultrasound cohort, with a difference of −18 percentage points (CI −31 to −5).

**Table 1:** Baseline characteristics of patients undergoing elective THA revision. Values are count (%) or as specified

Item	Fluoroscopy n = 117	Ultrasound n = 90
Age, median (IQR)	66 (57–73)	69 (56–77)
Female	58 (50)	50 (56)
Body mass index, median (IQR)	28 (24–31)	27 (24–32)
missing values	–	2 (2)
Reason for revision surgery		
(Suspected) PJI	53 (45)	49 (54)
Aseptic loosening	57 (49)	37 (41)
Other	7 (6)	4 (4)
PJI ^**a**^	54 (46)	25 (28)

IQR = interquartile range; THA = total hip arthroplasty.

a PJI (periprosthetic joint infection) diagnosis based on EBJIS (European Bone and Joint Infection Society) criteria after revision surgery.

Diagnostic accuracy could be assessed in 78/117 (67%) fluoroscopy-guided procedures and 53/90 (59%) ultrasound-guided procedures. The remaining procedures were dry taps (38 and 31, respectively) or had a laboratory sampling error (1 and 6, respectively). Diagnostic accuracy was also assessed for all 39 ultrasound-guided biopsies (Figure).

**Figure:**
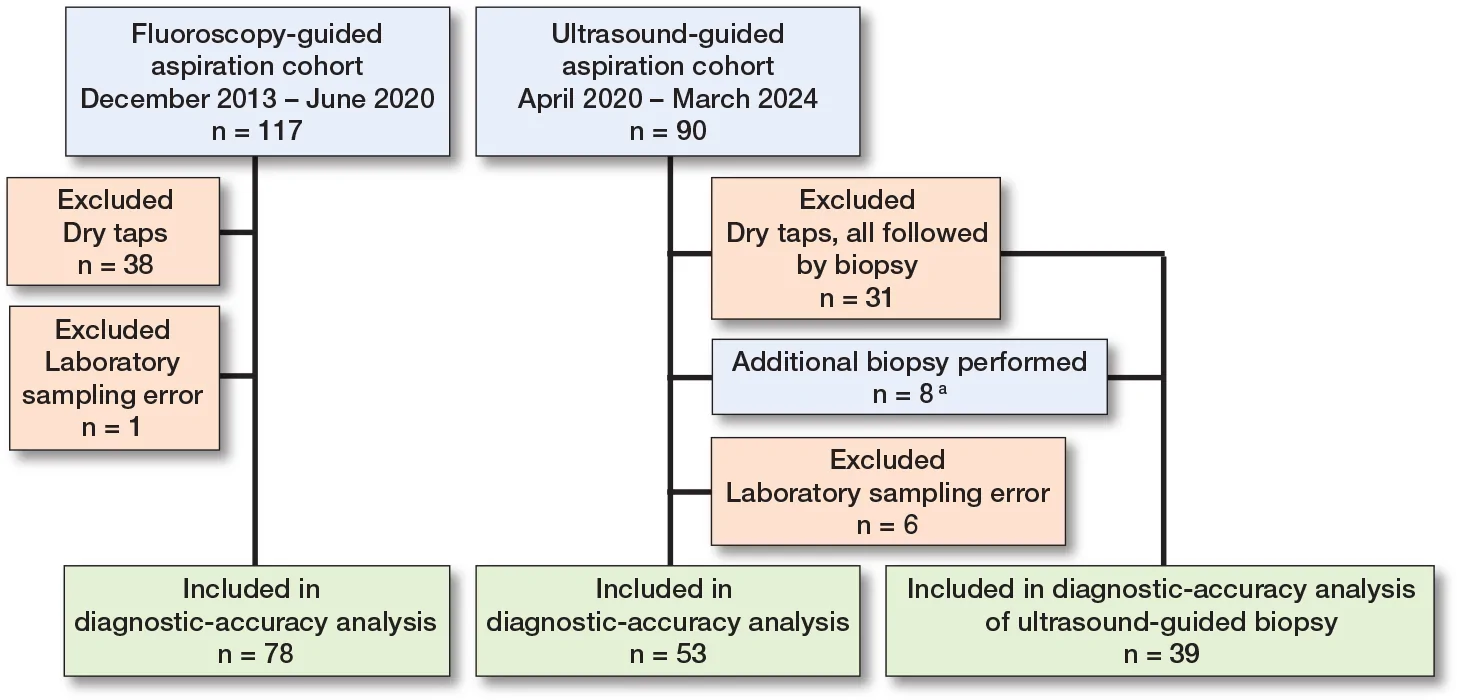
Study flow for procedural and diagnostic-accuracy analyses of image-guided hip aspiration and biopsy. Procedures were included in the aspiration diagnostic-accuracy analyses only when synovial fluid was obtained, the aspiration culture was interpretable, and the postoperative reference standard was available. In the ultrasound cohort, all 31 dry taps were followed by ultrasound-guided biopsy, and an additional biopsy was performed after 8 successful aspirations, resulting in 39 biopsies included in the diagnostic-accuracy analysis. The cohorts overlapped during the institutional transition period from April to June 2020. ^**a**^ Biopsy was performed after successful aspiration when additional diagnostic information was considered clinically relevant following consultation with the treating orthopedic surgeon.

### Procedural outcomes

Dry taps occurred in 38/117 (32%) fluoroscopy-guided and 31/90 (34%) ultrasound-guided procedures, corresponding to an absolute difference of 2 percentage points (CI –11 to 16). Synovial fluid volume was documented in 67/117 (57%) fluoroscopy-guided and 59/90 (66%) ultrasound-guided procedures. Among procedures with documented volume, median aspirated volume was 5 mL (IQR 3–10) in the fluoroscopy cohort and 4 mL (IQR 2–15) in the ultrasound cohort, with a Hodges–Lehmann estimate of the between-cohort difference of 1 mL (CI –1 to 2). No complications were recorded in the fluoroscopy cohort, whereas 2/90 (2%) procedures in the ultrasound cohort were associated with a complication, corresponding to an absolute difference of 2 percentage points (CI –2 to 9). The complications comprised 1 hematoma and 1 puncture-site leakage, both in patients who underwent additional biopsy (Table 2).

**Table 2:** Procedural outcomes of fluoroscopy-guided and ultrasound-guided aspiration. Values are count (%) or as specified

Item	Fluoroscopy n = 117	Ultrasound n = 90	Absolute difference, percentage points (CI)
Dry tap	38 (32)	31 (34)	2 (–11 to 16)
Volume aspirated, mL			
median (IQR)	5 (3–10)	4 (2–15)	1 (–1 to 2)
missing values	50 (43)	31 (34)	
Complications	0 (0)	2 (2) ^**a**^	2 (–2 to 9)

CI = 95% confidence interval; IQR = interquartile range.

a Complications consisted of 1 hematoma and 1 case of puncture site leakage; each occurred in a patient who underwent an additional ultrasound-guided biopsy.

### Diagnostic accuracy

In the fluoroscopy-guided accuracy cohort, aspiration culture yielded 16 true-positive, 3 false-positive, 18 false-negative, and 41 true-negative results. Corresponding counts in the ultrasound-guided cohort were 8, 3, 5, and 37, respectively (Table 3).

**Table 3:** 2 x 2 diagnostic tables of aspiration culture results compared with the reference standard. Values are count

Item	PJI ^a^	Aseptic failure	Total
Fluoroscopy-guided aspiration			
Aspiration culture positive	16	3	19
Aspiration culture negative	18	41	59
Total	34	44	78
Ultrasound-guided aspiration			
Aspiration culture positive	8	3	11
Aspiration culture negative	5	37	42
Total	13	40	53

a See Table 1.

Sensitivity was 16/34 (47%, CI 30–65) for fluoroscopy-guided aspiration and 8/13 (62%, CI 32–86) for ultrasound-guided aspiration. Specificity was 41/44 (93%, CI 81–99) and 37/40 (93%, CI 80–98), respectively. The exploratory between-cohort absolute difference was 14 percentage points (CI −17 to 42) for sensitivity and 0 percentage point (CI −14 to 12) for specificity (Table 4).

**Table 4:** Diagnostic accuracy of fluoroscopy-guided and ultrasound-guided aspiration

Item	Fluoroscopy % (CI)	Ultrasound % (CI)	Absolute difference, percentage points (CI)
Sensitivity	47 (30–65)	62 (32–86)	14 (–17 to 42)
Specificity	93 (81–99)	93 (80–98)	0 (–14 to 12)

CI = 95% confidence interval.

### Sensitivity analyses

When dry taps and laboratory sampling errors were classified as test-positive, the fluoroscopy-guided cohort yielded 36 true-positive, 22 false-positive, 18 false-negative, and 41 true-negative results. Corresponding counts in the ultrasound-guided cohort were 20, 28, 5, and 37, respectively (Supplementary Table S1). Sensitivity was 36/54 (67%, CI 53–79) in the fluoroscopy cohort and 20/25 (80%, CI 59–93) in the ultrasound cohort, corresponding to an absolute between-cohort difference of 13 percentage points (CI –9 to 32). Specificity was 41/63 (65%, CI 52–77) and 37/65 (57%, CI 44–69), respectively, with an absolute difference of –8 percentage points (CI –25 to 9) (Supplementary Table S2).

When dry taps and laboratory sampling errors were classified as test-negative, the fluoroscopy-guided cohort yielded 16 true-positive, 3 false-positive, 38 false-negative, and 60 true-negative results. Corresponding counts in the ultrasound-guided cohort were 8, 3, 17, and 62, respectively (Supplementary Table S3). Sensitivity was 16/54 (30%, CI 18–44) and 8/25 (32%, CI 15–54), respectively, corresponding to an absolute difference of 2 percentage points (CI –18 to 25). Specificity was 60/63 (95%, CI 87–99) and 62/65 (95%, CI 87–99), with an absolute difference of 0 percentage points (CI –9 to 9) (Supplementary Table S4).

### Ultrasound-guided biopsy

Ultrasound-guided synovial biopsy was performed in 39 patients: 31 after a dry tap and 8 despite successful aspiration when additional diagnostic information was considered clinically relevant. Tissue culture yielded 5 true-positive, 3 false-positive, 9 false-negative, and 22 true-negative results (Table 5). Sensitivity was 5/14 (36%, CI 13–65) and specificity was 22/25 (88%, CI 69–97) (Table 6). In all 5 true-positive biopsy cases the same microorganism isolated from at least 2 biopsy specimens was also identified in the intraoperative tissue cultures (Supplementary Table S5).

**Table 5:** Ultrasound-guided synovial biopsy culture results compared with the postoperative reference standard. Values are count

Biopsy culture result	PJI ^a^	Aseptic failure	Total
≥ 2 positive samples with same microorganism	5	3	8
Negative or single positive sample ^**b**^	9	22	31
Total	14	25	39

a See Table 1.

b Biopsy cultures were considered positive if the same microorganism was isolated from at least 2 of 5 specimens. A single positive specimen was classified as a negative biopsy culture for the diagnostic accuracy analysis.

**Table 6:** Diagnostic accuracy of ultrasound-guided synovial biopsy cultures. Values are percentages with exact binomial 95% confidence intervals (CI).

Diagnostic accuracy	% (CI)
Sensitivity	36 (13–65)
Specificity	88 (69–97)

Sensitivity was calculated among 14 patients with infection and specificity among 25 patients without infection.

## Discussion

The aim of our exploratory study was to describe procedural outcomes and explore diagnostic accuracy across sequential real-world cohorts undergoing ultrasound-guided or fluoroscopy-guided hip aspiration. As a secondary exploratory objective, we described the diagnostic accuracy after ultrasound-guided biopsy. We found that fluoroscopy-guided to ultrasound-guided hip aspiration was not accompanied by a lower observed dry-tap proportion: approximately one-third of procedures were dry taps in both cohorts. Among procedures with an interpretable aspiration culture and an available postoperative reference standard, both techniques showed high specificity, but lower sensitivity estimates with substantial uncertainty. No complications were recorded in the fluoroscopy cohort; 2 events occurred in the ultrasound cohort, both in patients who underwent biopsy. These findings suggest broadly comparable procedural outcomes but do not establish equivalence, superiority, or comparative safety.

Comparative evidence remains limited but suggests higher aspiration success and diagnostic performance with ultrasound than with fluoroscopy [11–13]. A low dry-tap rate has also been reported with an ultrasound protocol incorporating needle redirection and saline lavage [14]. Differences in patient selection, aspiration technique, use of lavage, dry-tap definitions, reference standards, and analysis denominators limit direct comparison between these studies and the present cohorts. The present study adds a real-world evaluation of an institutional transition, with detailed reporting of procedural denominators, diagnostic performance, and sensitivity analyses, but should be regarded as exploratory.

The dry-tap proportions in the present study were similar between techniques and consistent with previous studies, which also indicate that dry taps do not exclude PJI and that synovial testing after lavage may be less sensitive [15,16]. In the present study, sensitivity estimates were conditional on obtaining an interpretable culture with substantial uncertainty around the difference between techniques. Extreme-case analyses illustrated how classifying dry taps and laboratory sampling errors as positive or negative tests affected sensitivity estimates. High specificity supports the diagnostic value of a positive culture when interpreted in the clinical context, whereas neither a negative culture nor a dry tap reliably excludes PJI. Potential explanations for false-negative cultures include limited sampling, recent antibiotic exposure, and biofilm-associated infection, but these factors could not be evaluated reliably. Predictive values were not emphasized because they depend on prevalence, which differed between cohorts.

In the ultrasound cohort, biopsy was performed after all dry taps and selectively after successful aspirations. Limited evidence suggests that a positive biopsy culture may help confirm PJI after a dry tap, whereas low sensitivity limits the value of a negative result for excluding infection [8,17]. Indications for biopsy after successful aspiration were not prespecified, and no corresponding biopsy strategy was available in the fluoroscopy cohort. The present study therefore cannot determine the incremental diagnostic value of biopsy or compare an aspiration-plus-biopsy pathway with aspiration alone.

### Limitations

First, the retrospective, single-center design is susceptible to selection bias and limits generalizability. Complications were identified retrospectively without standardized active surveillance, and the sample size provided limited precision for comparing rare adverse events. The few recorded complications occurred only among patients who underwent biopsy in the ultrasound cohort. These findings do not establish comparative safety, and differences in workflow and biopsy use prevent attribution of the observed complications to imaging modality alone. Second, the sequential cohorts spanned different calendar periods. Changes in diagnostic pathways, microbiological practice, aspiration indications, and operator experience may therefore have introduced temporal bias. A lower logistical threshold and broader use of aspiration in the later period may partly explain the lower PJI prevalence in the ultrasound cohort. Third, the cohorts were unpaired and the reported differences were unadjusted descriptive contrasts. Fourth, diagnostic accuracy was based on complete cases and excluded dry taps and laboratory sampling errors. Finally, the diagnostic-accuracy samples were small, resulting in wide confidence intervals and limiting additional analyses, making the conclusions less strong.

### Conclusion

We found that the transition from fluoroscopy-guided to ultrasound-guided hip aspiration was not accompanied by a lower observed dry-tap proportion: approximately one-third of procedures were dry taps in both cohorts. Among procedures with an interpretable aspiration culture and an available postoperative reference standard, both techniques showed high specificity, but lower sensitivity estimates with substantial uncertainty. In perspective, ultrasound guidance may offer practical workflow advantages by avoiding ionizing radiation and operating room use, but the sequential, non-contemporaneous design does not establish equivalence, superiority, or comparative safety.

### Supplementary data

Tables S1–S5 are available as supplementary data on the article home page, doi: 10.2340/17453674.2026.46931

## Supplementary Material



## Notes

The study was designed by TS and WR. TS collected the data. TS, KM, GH, and WR analyzed the data. TS and KM prepared the initial manuscript. GH, JS, JG, BS, and WR critically revised the manuscript, and approved the final version.

Handling co-editors: Keijo Mäkelä and Robin Christensen

*Acta* thanks Henrik Bodén and other anonymous reviewers for help with peer review of this manuscript.
