# Supplementary material for: Ultrasound- and fluoroscopy-guided hip aspiration before revision total hip arthroplasty: procedural outcomes and diagnostic accuracy in sequential cohorts
Source: Acta Orthop. 2026 Oct 8;97:734–9. doi: 10.2340/17453674.2026.46931 (PMC13646778; doi:10.2340/17453674.2026.46931)
Supplement: Supplementary file 1 [file ActaO-97-46931-s1.pdf]

Supplementary files

Supplementary Table S1. Two-by-two diagnostic tables of aspiration culture results including dry taps and laboratory sampling errors classified as test-positives, compared with the reference standard

	PJI [a]	Aseptic failure	Total
**Fluoroscopy-guided aspiration**			
Aspiration culture positive	36	22	58
Aspiration culture negative	18	41	59
**Total**	**54**	**63**	**117**
**Ultrasound-guided aspiration**			
Aspiration culture positive	20	28	48
Aspiration culture negative	5	37	42
**Total**	**25**	**65**	**90**

[a] PJI (periprosthetic joint infection) diagnosis was based on EBJIS (European Bone and Joint Infection Society) criteria after revision surgery.

Supplementary Table S2. Diagnostic accuracy of fluoroscopy-guided and ultrasound-guided aspiration with dry taps and laboratory sampling errors classified as test-positives

	Fluoroscopy % (CI)	Ultrasound % (CI)	Absolute difference percentage points (CI)
**Sensitivity**	67 (53–79)	80 (59–93)	13 (–9 to 32)
**Specificity**	65 (52–77)	57 (44–69)	–8 (–25 to 9)

CI = 95% confidence intervals.

Supplementary Table S3. Two-by-two diagnostic tables of aspiration culture results including dry taps and laboratory sampling errors classified as test-negatives, compared with the reference standard.

	PJI [a]	Aseptic failure	Total
**Fluoroscopy-guided aspiration**			
Aspiration culture positive	16	3	19
Aspiration culture negative	38	60	98
**Total**	**54**	**63**	**117**
**Ultrasound-guided aspiration**			
Aspiration culture positive	8	3	11
Aspiration culture negative	17	62	79
**Total**	**25**	**65**	**90**

[a] PJI (periprosthetic joint infection) diagnosis was based on EBJIS (European Bone and Joint Infection Society) criteria after revision surgery

Supplementary Table S4. Diagnostic accuracy of fluoroscopy-guided and ultrasound-guided aspiration with dry taps and laboratory sampling errors classified as test-negatives

	Fluoroscopy % (CI)	Ultrasound % (CI)	Absolute difference percentage points (CI)
**Sensitivity**	30 (18–44)	32 (15–54)	2 (–18 to 25)
**Specificity**	95 (87–99)	95 (87–99)	0 (–9 to 9)

CI = 95% confidence intervals.

Supplementary Table S5. Microbiological results of ultrasound-guided biopsy cultures and corresponding intraoperative cultures in patients with a positive biopsy culture (n = 8)

No.	Microorganisms identified in biopsy cultures	Microorganisms identified in intraoperative cultures
1	Group B β-hemolytic streptococci	Group B β-hemolytic streptococci
2	*Staphylococcus aureus*	*Staphylococcus aureus*
3	*Staphylococcus epidermidis*	No growth
4	*Candida glabrata*	*Candida glabrata*
5	*Staphylococcus epidermidis*	No growth
6	*Staphylococcus caprae*	*Staphylococcus caprae* and *Staphylococcus aureus*
7	*Staphylococcus epidermidis*	*Staphylococcus epidermidis*
8	*Staphylococcus aureus*	No growth
